# Physical Disorders and Poor Self-Rated Health in Adults Living in Four Latin American Cities: A Multilevel Approach

**DOI:** 10.3390/ijerph17238956

**Published:** 2020-12-02

**Authors:** Camila Vaz, Amanda Cristina Andrade, Uriel Silva, Daniel Rodríguez, Xize Wang, Kari Moore, Amélia Augusta Friche, Ana Victoria Diez-Roux, Waleska Teixeira Caiaffa

**Affiliations:** 1 Department of Physical Therapy, Federal University of Juiz de Fora, Rua São Paulo 745, Governador Valadares 35010-180, Brazil; 2 Belo Horizonte Observatory for Urban Health, Avenida Alfredo Balena 190, Belo Horizonte 30130-100, Brazil; amandasouza_est@yahoo.com.br (A.C.A.); urielmoreirasilva@gmail.com (U.S.); gutafriche@gmail.com (A.A.F.); caiaffa.waleska@gmail.com (W.T.C.); 3 Institute of Public Health, Federal University of Mato Grosso, Avenida Fernando Corrêa 2367, Cuiabá 78060-900, Brazil; 4 Department of City and Regional Planning and Institute for Transportation Studies, University of California, 228 Wurster Hall, Berkeley, CA 94720, USA; danrod@berkeley.edu; 5 Department of Real State, National University of Singapore, 4 Architecture Drive, Singapore 117566, Singapore; wang.xize@nus.edu.sg; 6 Urban Health Collaborative, Dornsife School of Public Health, Drexel University, 3215 Market Street, Philadelphia, PA 19104, USA; kam642@drexel.edu (K.M.); avd37@drexel.edu (A.V.D.-R.); 7 Faculty of Medicine, Federal University of Minas Gerais, Avenida Alfredo Balena 190, Belo Horizonte 30130-100, Brazil

**Keywords:** self-rated health, physical disorder, environment, health status, Latin America, urban health, multilevel analysis

## Abstract

Considering that urban environments may affect self-rated health through behavioral and psychosocial mechanisms, the aim of this study was to investigate the association between self-rated health and perceived urban environment characteristics among adults living in four Latin American cities. Data is from a population-based survey by Development Bank of Latin America, encompassing adults between 20 and 60 years old in Buenos Aires, Lima, Mexico City, and Panama City. Self-rated health was measured using a single question and the response options were categorized as poor and good. The explanatory variables were empirical Bayes estimates of self-reported area physical disorder, social disorder, access to services, and access to leisure spaces derived from the survey. The covariates were: individual age, sex, education, wealth index, and length of residency in the neighborhood; and an area social environment index. Multilevel logistic regressions with two levels (individual and sub-city areas) were fitted. Poor self-rated health was reported by 34.73% (95% CI: 33.17 to 36.29) of the participants and was associated with physical disorder (OR = 1.16 per SD; 95% CI: 1.02 to 1.32). Our findings suggest that public policies to promote population health should consider area urban environment factors, especially those associated with disorder.

## 1. Introduction

Research shows that self-rated health is a significant predictor of morbidity and mortality as well as health care utilization, even after controlling for individual-level characteristics [1,2,3,4]. This valid, reliable, simple, and easily administered question is commonly used to characterize health in population surveys [1]. Self-rated health is a multidimensional construct that can be examined in relation to various factors [1,2,3].

Previous studies have investigated the determinants of self-rated health with an emphasis on either personal or family traits, such as educational attainment, employment status and family structure [5,6,7,8]. More recently, considerable attention has been given to the impacts of neighborhood characteristics or urban environments on health [9]. Urban environment features may affect health through a multiplicity of processes including influencing health-related behaviors through features such as walkability, the location of parks and green spaces, and access to and advertising for different types of foods. Other mechanisms may include community stressors such as violence or lack of safety and the availability of social connections and social support mechanisms [10,11,12,13,14,15,16].

A growing literature has investigated associations between urban environments and health outcomes in high income countries [17]. However, this question has not been widely explored in Latin America, a region with high levels of urbanization and the highest socioeconomic inequality in the world [18,19]. Specific features of Latin American cities including the ways in which urbanization has occurred and the specific patterns of residential segregation and inequality present in the region may affect the ways which urban environments affect health [20].

A recent systematic review of the impact of urban environments on self-rated health in Latin America found few studies on this subject and suggested that heterogeneities among countries indicate the need for additional studies of the largest urban settings of the region [11]. We used survey data from a sample of adults residing in capitals of four large Latin American countries: Argentina (Buenos Aires), México (México City), Peru (Lima), Panamá (Panamá City), to investigate the association of perceived urban environment characteristics with self-rated health. This study contributes to the literature in three ways. First, standardized data collection enabled us to use comparable outcome and environmental exposures in the four cities. Second, we used state-of-the-art statistical methods to derive measures of important features of urban environments and used multilevel analysis to simultaneously examine individual and area-level characteristics. Third, we linked survey data to other census-based area measures to allow for additional adjustment for areas socioeconomic features.

## 2. Materials and Methods

### 2.1. Study Design

Data were obtained from a population-based survey carried out by the Development Bank of Latin America (CAF survey), from November 2016 to January 2017. The CAF survey used a semi-probabilistic, multi-stage stratified sampling approach described elsewhere [21]. For each selected household, one individual was interviewed. The survey included 12,905 participants, between 20 and 60 years old, in 11 Latin American cities: Bogota (COL), Buenos Aires (ARG), Caracas (VEN), Fortaleza (BRA), La Paz (BOL), Lima (PER), Mexico City (MEX), Montevideo (URY), Panama City (PAN), Quito (ECU) and Sao Paulo (BRA). Data were collected through in-person interviews. A questionnaire was administered to collect respondent demographic and socioeconomic information, information on self-reported health and self-perceived neighborhood characteristics, as well as a set of characteristics of the household [21].

Our analyses focused only on cities where sub-city level data is available from the Salud Urbana en América Latina (SALURBAL), Urban Health in Latin America Project. SALURBAL is an international collaboration that studies how urban environments and urban policies impact the health of city residents throughout Latin America [22]. In SALURBAL, a city is defined as a single administrative unit (e.g., municipio) or combination of adjacent administrative units (e.g., several municipios) that are part of the urban extent as determined from satellite imagery [22]. Four of the 11 cities surveyed (Caracas, La Paz, Montevideo, and Quito) were excluded because their respective countries are not part of the SALURBAL study. In the seven remaining cities, respondents’ residential addresses were georeferenced to “sub-city” units within the cities. “Sub-city” is defined in the SALURBAL Project as an administrative unit (e.g., comuna, municipio) nested within a city [23]. This was the smallest geographic unit to which survey data could be linked. These sub-city units are henceforth referred to as sub-city areas.

Of the seven cities in SALURBAL, three (Bogota, Fortaleza and Sao Paulo) contained respondents in only one “sub-city” unit and were excluded from the sample. Therefore, the final sample consisted of four cities: Buenos Aires, Lima, Mexico City, and Panama City. These four cities had a large number of “sub-city” units (Table 1). In Buenos Aires, the CAF survey conducted an oversampling of the informal settlement areas, including 550 respondents [21]. Because samples from the other cities did not include informal settlements, these participants were excluded from the analysis sample. We included a total of 3588 respondents nested in 139 areas with a median number of 19 respondents for area (Table 1).

The SALURBAL study protocol was approved by the Drexel University Institutional Review Board with ID #1612005035.

### 2.2. Outcome

The response variable was self-rated health, measured with the question “In general, would you say your health is ...?” Response options were categorized as 1 = poor (bad and fair) and 0 = good (good).

### 2.3. Explanatory Variables

Perceived characteristics of the environment are divided into four domains: Physical Disorder, Social Disorder, Access to Services, and Access to Leisure Spaces, were assessed in the survey.

The Physical Disorder scale was constructed based on the following questions: “Do any of the following situations occur within three blocks or less of your home?: (1) Abandoned buildings, houses or lots; (2) Buildings, houses or lots taken illegally or invaded; (3) Garbage dumps; (4) Poorly lit streets; and (5) Drug purchases and use”. The answer choices to these questions were yes and no.

The Social Disorder scale was constructed from the following Likert-type questions: “On a scale of 1 to 5, where 1 means “never” and 5 means “always”, how often would you say that the following situations occur in your block?: (1) Acts of aggression and/or offenses; (2) Indigence/Begging; (3) Gang activity; (4) Prostitution; and (5) Conflict between neighbors”. The answer choices to these questions were: never, rarely, sometimes, almost always and always.

The Access to Services scale was constructed using the following question: “How far (in walking time) are the following establishments from your home (consider the closest)?: (1) Hospitals or health centers; (2) Public primary or secondary schools; (3) Gardens or child care centers for children under 5 years; and a (4) Police station” and “How long do you need to walk from your home to access the following modes of transportation?: (5) Bus, or similar transport (*metrobus*, *Transmilenio*)”; and (6) Metro/Subway. The answer choices to these questions were: less than 10 min, between 10 and 30 min and more than 30 min.

The Access to Leisure Spaces scale was constructed using the following question: “How long would it take you to walk to the closest of the following services or public establishments?: (1) Parks, plazas or green areas; (2) Public libraries or cultural centers; and (3) Community sports or recreation centers”. The answer choices to these questions were: less than 10 min, between 10 and 30 min and more than 30 min.

The number of participants that did not answer at least one of the items was 610 (17%) for the Physical Disorder scale, 182 (5%) for the Social Disorder scale, 1291 (36%) for the Access to Services scale and 751 (21%) for the Access to Leisure Spaces. In order to avoid the nonnegligible loss of participants that would be incurred by simply excluding the non-respondents from the sample, multiple logistic imputation (conditional on sex, age and a fixed intercept for each city, with Buenos Aires as reference) was performed for each missing item response. All of the participants answered at least one question per scale.

Scores for each scale were created by summing the responses after assigning ordinal values to response options. Higher values indicate more social disorder, more physical disorder and longer distances to service or leisure places. Internal consistency for each scale was assessed using Cronbach’s Alpha. The questions had an acceptable internal consistency, with Cronbach’s Alpha ranging from 0.62 to 0.76 (Table S1).

We then used multilevel models to estimate the area level measurement properties of the scales and to derive empirical Bayes estimates of values for each area by combining the responses of all respondents in the same area [12,24,25]. In the model, level 1 corresponded to item responses within individuals; level 2 corresponded to individuals nested within areas; and level 3 corresponded to the area. We pooled the four cities together and adjusted for city to estimate the four area scales. The sample intra-area correlation coefficients (ICC) varied between 0.16 and 0.30. The area-level reliability of the scales varied between 0.95 and 0.98 (Table S1). The scales were standardized using z-scores, with mean equal to 0 and standard deviation of 1. A higher score indicates a greater presence of the attribute in the neighborhood.

Spearman’s correlation coefficients were used to estimate correlations between neighborhood scales. Correlations between the scales were weak and moderate [26], varying in magnitude from 0.08 to 0.45, except the correlation between the Access to Services and Access to Leisure Spaces which was 0.77 (data not shown).

### 2.4. Covariates

Other covariates included were individual characteristics and a social environment index at the area level, defined below.

The individual characteristics were: length of residency in the area (years), age (years), sex (female and male), education (primary school or less; high school degree, technical, some college; and university degree or higher), and a Wealth Index (scale ranges from 0–100, with a higher score denoting higher household wealth).

The Wealth Index is an asset-based index that measures a household’s long-term economic status, and that has been validated for use in low and middle-income countries. The index is based on: (a) ownership of consumer durables (television, refrigerator, cell phone, car, bicycle, cheap utensils and expensive utensils); (b) access to basic services (electricity and water sources); and (c) housing characteristics (quality of floor material, toilet facility and numbers of sleeping rooms) [27].

The social environment index at the area level was obtained from SALURBAL data and computed using data from each of the countries’ censuses for each city (Buenos Aires, Mexico City and Panama City’s data is from 2010 and Lima’s is from 2002). The z-scores of each of the following variables were calculated: (a) proportion of households with piped water access inside the dwelling; (b) proportion of households connected to a public sewage network; (c) proportion of households with more than 3 people per room (reversed); and (d) proportion of the population aged 25 or older who completed primary education or above. The index was then defined as the mean of the z-scores, with a higher score indicating a “better” social environment (i.e., more access to sanitation, higher education, etc.). All variables used to create this index were harmonized by the SALURBAL Project’s team in order to guarantee comparability across countries [23].

### 2.5. Statistical Analysis

Descriptive analyses were performed using frequency distributions, means and standard deviation (SD). Kruskal-Wallis or chi square test were used as appropriate to compare variables across cities and by levels of self-reported health. Multilevel logistic regressions were used to estimate the association between poor self-rated health and the explanatory variables. These models had two levels: individual and sub-city area, with a fixed effect for each city.

The first model included only the area-level scales (bivariate analyses in separate models). The second model adjusted each scale for individual-level covariates (length of residency, age, sex, education, and Wealth Index). The third model added the social environment index. Finally, the fourth model, the full model, included all the scales and all the covariates (individual and contextual) together. Multiplicative interactions between education and area scales and between Wealth Index and area scales were tested. The analyses were conducted using Stata, version 12.0 (StataCorp LP, College Station, TX, USA).

## 3. Results

The total sample across the four cities was 3606 participants; 18 were excluded due to lack of information regarding the outcome variable. The remaining 3588 participants were nested in a total of 139 sub-city areas and the median number of participants per area was 19, ranging from 1 to 178. Sample characteristics by city are shown in Table 1. The overall proportion of poor self-rated health was 34.7% (95% CI: 33.2–36.3). Lima (47.2%) had the highest proportion of poor self-rated health among residents, followed, sequentially, by Mexico City (36.9%), Panama City (31.4%) and Buenos Aires (22.1%). The study population was 52.4% female and the mean age was 37.58 years (SD = 11.76). The majority of participants had a high school or technical degree (49.01%) and the mean Wealth Index was 75.31 (SD = 15.78) (Table 1).

Distributions by age and sex did not differ substantially across cities (Table 1). However, the Lima and Panama City samples had higher levels of education than the Buenos Aires and Mexico City samples. Buenos Aires and Panama City had higher scores of Wealth Index than the Lima and Mexico City. The mean of Physical Disorder was higher in Buenos Aires and Mexico City than in the other cities; the mean of Social Disorder scale was highest in Lima and Panama City; and the mean of Access to Services and Access to Leisure Spaces scales were highest in Buenos Aires and Panama City. The Social Environment Index had higher values in Mexico City and Panama and lower values in Buenos Aires and Lima (Table 1).

Individuals who reported poor health were more likely to be female, older, less educated and had a lower mean Wealthy Index score than those who reported good health. The area-level Physical and Social Disorder scales had slightly higher means among people who reported poor health. The Social Environment Index was lower in those who reported poor health compared to those who reported good health. (Table 2).

Table 3 shows associations of area level variables with self-rated health. In unadjusted models (model 1), people who lived in areas with more perceived Physical and Social Disorder were more likely to report poor health (OR: 1.21 per increase in one SD unit; 95% CI: 1.10–1.34 and OR: 1.14 per increase in one SD unit; 95%CI: 1.03–1.27, respectively). Only the association between Physical Disorder scale and poor health persisted and was slightly attenuated after adjustment for the individual-level covariates (model 2), OR: 1.18 per increase in one SD unit; 95% CI: 1.06–1.30. With further adjustment for the social environment index (model 3) this association was only slightly attenuated (OR: 1.15 per increase in one SD unit; 95% CI: 1.03–1.28). Finally, after adjustment for all individual and area-level covariates and all area scales, the full model, the Physical Disorder scale remained significantly associated with poor health (OR: 1.16 per increase in one SD unit; 95% CI: 1.02–1.32), indicating that people who lived in areas with more perceived Physical Disorder were more likely to report poor health. None of the interactions terms we tested were statistically significant. As a sensitivity analyses, we also constructed the area-level scales without imputation and assessed their association with self-rated health (Table S2). Compared to the results shown in Table 3, conditional imputation only had a marginal effect on the point estimates of the associations between self-rated health and the scales but had a significant effect on their precision.

## 4. Discussion

We investigated whether perceived urban environment characteristics were associated with self-rated health among adults living in four Latin American cities (Buenos Aires, Lima, Mexico City and Panama City). Our analysis showed that people living in areas with higher Physical Disorder, an indicator that aggregates features such as abandoned buildings, houses or lots; buildings, houses or lots taken illegally or invaded; garbage dumps; poorly lit streets; and drug purchase and use, were more likely to report poor health, even after controlling for individual and other area-level characteristics.

These findings are in agreement with studies in countries of the global North and other countries in Latin America that showed an association between perceived physical disorder and self-rated health [12,28,29,30,31]. Poortinga and colleagues found that urban environment characteristics, such as litter and rubbish; vandalism; and discarded needles and syringes, had the strongest associations with poor health at the neighborhood level in Wales, United Kingdom [28]. In Brazil, researchers observed that perceived physical disorder, such as garbage; presence of graffiti; vacant lots; vandalism; street lighting, were also associated with poor self-rated health [12,31].

Physical disorder is related to the deterioration of the urban landscape and may also indicate of a lack of formal and informal social control [30,31]. Many individuals that live in areas under such conditions may find life threatening and difficult, making the development of neighborhood trust, attachment, and participation in community life more challenging [32,33,34]. Within a disordered urban environment, many neighbors may be reluctant to venture outside, discouraging healthful outdoor activities, such as walking, and reducing their ability to form ties and observe positive neighborhood interaction [33,35]. It has been argued that the psychosocial process of weakened social cohesion may contribute to negative health related outcomes and risky health-related behaviors in environments with high physical disorder [36,37,38,39,40].

Our study did not find any associations between self-rated health and other area scales, such as Social Disorder, Access to Services and Access to Leisure Spaces in fully adjusted models. Association observed for social disorder in the unadjusted model disappeared when individual-level covariates were adjusted for. Other studies have found that quality of services, mobility, public facilities, safety from traffic, violence, presence of parks, street noise, income, overcrowding, and the water, sewage network, electricity, and garbage collection services were related to health status [12,15,31,41,42,43]. It is possible that many of these elements are better captured in the physical disorder scale that we used. The correlation between the physical and the social disorder scale on our study was 0.45.

It is important to highlight that we used participant reports to derive measures of the urban environment. The advantage of using urban environment perceptions is that it captures information on particular features of the physical and social environment that cannot be obtained from secondary data or other sources such as systematic social observation. The use of area-level aggregated survey responses to characterize areas and the empirical Bayesian estimation method adopted for the construction of the scales allows for an overall reduction in measurement error due to individual subjectivity, since responses across multiple respondents within an area are combined [25]. An important limitation of our study is that we pooled responses across large areas thus missing smaller scale heterogeneity across neighborhoods within areas [24,44]. In addition, we did not include other more specific measures of physical or social environments (such as access to healthy foods and recreational facilities, air pollution exposures, or violence) all of which may affect health and either mediate or confound the effects of physical disorder on health.

Although the measure of self-rated health is an important indicator of population health and is correlated with objectively measured health outcomes [2,3,45], the question used in this study had only three answer options: bad, fair and good (*mala*, *regular* and *buena* in Spanish). Other studies use a version with five response options (very good, good, fair, poor and very poor) limiting the comparability of our findings to these studies. Another limitation pertains to the translation of the fair category as regular in the survey. It has been shown that some Spanish-language respondents may answer regular when they actually mean to rate their health in more positive terms than conveyed by the English term fair [46].

The cross-sectional design of our study does not allow us to draw causal inferences and confounding remains a possibility. The samples were not representative of the full cities. The dataset had about 40% of missing data on income but we addressed this by creating instead a wealth index that measures a household’s long-term economic status. Although the individuals included and excluded in this study sample were comparable, they may be systematically different in unobserved ways that may be associated with the exposures and the outcome. Similarly, because we estimated many models it is more likely that some results appear as statistically significant due to chance. Finally, another limitation is associated with the use of administrative units (area-level in our study) that are large and therefore may not capture the most etiologically relevant residential context. Identifying the correct geographical level of analysis is important, as misspecification may have implications for the study outcomes [34]. Nevertheless, even the large units we used reveal significant heterogeneity in urban environments within cities.

## 5. Conclusions

Using a relatively large sample in four Latin American cities from four different countries we showed that higher levels of physical disorder were related to higher odds of poor self-rated health. These results were observed even after controlling for individual/household demographics and socioeconomic variables, and other area social environment features. The reasons for these associations need to be fully understood, but their presence highlights the importance of urban physical environments to health in rapidly urbanizing low and middle income countries. Further studies with longitudinal designs and the use of objective measures of the environment, such as systematic social observation, are needed to corroborate these results.

## Figures and Tables

**Table 1:** Sample, individual and area characteristics, total and by city, CAF Survey, 2016–2017.

Variables	Total	Buenos Aires	Lima	Mexico City	Panama City	*p*-Value
Sample characteristics						
Number of participants	3588	1000	995	997	596	-
Number of areas	139	41	34	26	38	-
Participants by area						-
Median	19	21	19.5	26.5	12	
25th Percentile	11	16	12	15	9	
75th Percentile	31	29	41	48	19	
Minimum	1	1	5	1	1	
Maximum	178	130	106	178	84	
Individual Characteristics						
Poor self-rated health, *n* (%)	1246 (34.73)	221 (22.10)	410 (47.24)	368 (36.91)	187 (31.38)	<0.001
Age, m (SD)	37.58 (11.76)	38.26 (11.73)	36.33 (11.58)	37.95 (11.66)	37.91 (12.12)	<0.001
Sex, *n* (%)						0.714
Male	1708 (47.60)	478 (47.80)	476 (47.84)	461 (46.24)	293 (49.16)	
Female	1880 (52.40)	522 (52.20)	519 (52.16)	536 (53.76)	303 (50.84)	
Education, *n* (%) ^a^						<0.001
Primary school or less	1472 (41.03)	501 (50.10)	258 (25.93)	527 (52.86)	186 (31.21)	
High school	1757 (48.97)	446 (44.60)	639 (64.22)	383 (38.42)	289 (48.49)	
University degree or more	356 (9.92)	53 (5.30)	98 (9.85)	84 (8.43)	121 (20.30)	
Wealth Index, m (SD) ^b^	75.31 (15.78)	80.88 (12.98)	70.97 (16.63)	74.15 (15.88)	75.13 (17.73)	<0.001
Length of residency, m (SD) ^c^	18.39 (14.33)	17.20 (14.20)	18.01 (13.85)	21.50 (14.47)	15.83 (14.25)	<0.001
Area characteristics						
Physical Disorder, m (SD)	1.32 (0.09)	1.34 (0.09)	1.31 (0.07)	1.35 (0.09)	1.26 (0.06)	<0.001
Social Disorder, m (SD)	2.08 (0.33)	1.88 (0.25)	2.22 (0.25)	2.20 (0.34)	1.96 (0.32)	<0.001
Access to Services, m (SD)	2.38 (0.19)	2.52 (0.15)	2.45 (0.11)	2.25 (0.17)	2.23 (0.11)	<0.001
Access to Leisure Spaces, m (SD)	2.26 (0.17)	2.36 (0.17)	2.31 (0.12)	2.21 (0.16)	2.12 (0.13)	<0.001
Social Environment Index, m (SD)	0.42 (0.49)	0.34 (0.47)	0.18 (0.52)	0.64 (0.30)	0.58 (0.54)	<0.001

m = mean; SD = standard deviation; ^a^ 3 missing; ^b^ 178 missing; ^c^ 2 missing.

**Table 2:** Individual and area characteristics by self-rated health status CAF Survey, 2016–2017.

Variables	Self-Rated Health		*p*-Value
	Good	Poor	
**Individual Characteristics**			
Age, m (SD)	35.70 (11.28)	41.12 (11.83)	<0.001
Sex, *n* (%)			<0.001
Male	1178 (50.30)	530 (42.54)	
Female	1164 (49.70)	716 (57.46)	
Education, *n* (%) ^a^			<0.001
Primary school or less	875 (37.36)	597 (47.91)	
High school	1203 (51.37)	554 (44.46)	
University degree or more	262 (11.19)	94 (7.54)	
Wealth Index, m (SD) ^b^	77.46 (15.15)	71.23 (16.14)	<0.001
Length of residency, m (SD) ^c^	17.47 (13.77)	20.13 (15.18)	<0.001
City of residency, *n* (%)			<0.001
Buenos Aires	779 (33.26)	221 (17.74)	
Lima	525 (22.42)	470 (37.72)	
Mexico City	629 (26.86)	368 (29.53)	
Panama City	409 (17.46)	187 (15.01)	
**Area characteristics**			
Physical Disorder, m (SD)	1.32 (0.09)	1.33 (0.08)	0.004
Social Disorder, m (SD)	2.05 (0.33)	2.13 (0.32)	<0.001
Access to Services, m (SD)	2.38 (0.19)	2.37 (0.18)	0.121
Access to Leisure Spaces, m (SD)	2.26 (0.18)	2.26 (0.16)	0.258
Social Environment Index, m (SD)	0.45 (0.49)	0.36 (0.50)	<0.001

m = mean; SD = standard deviation; ^a^ 3 missing; ^b^ 178 missing; ^c^ 2 missing.

**Table 3:** Odds ratios of self-rated health associated with the area level characteristics before and after adjustment for covariates, CAF Survey, 2016–2017.

Variables	Model 1 ^a^		Model 2 ^b^		Model 3 ^c^		Model 4 ^d^	
	OR (95% CI)	*p*-Value	OR (95% CI)	*p*-Value	OR (95% CI)	*p*-Value	OR (95% CI)	*p*-Value
Physical Disorder	1.21 (1.10–1.34)	˂0.001	1.18 (1.06–1.30)	0.002	1.15 (1.03–1.28)	0.012	1.16 (1.02–1.32)	0.021
Social Disorder	1.14 (1.03–1.27)	0.013	1.11 (0.99–1.23)	0.062	1.09 (0.98–1.21)	0.102	1.02 (0.90–1.15)	0.769
Access to Services	0.99 (0.87–1.13)	0.902	1.06 (0.93–1.21)	0.402	1.12 (0.98–1.28)	0.110	1.06 (0.89–1.25)	0.513
Access to Leisure Spaces	0.96 (0.86–1.08)	0.529	1.03 (0.92–1.15)	0.656	1.08 (0.96–1.21)	0.215	1.09 (0.94–1.27)	0.259

SD = standard deviation; N individual level = 3405; N contextual level = 139; ^a^ Bivariate analyses between poor self-rated health and neighborhood scales; ^b^ Model adjusted for individual covariates (length of residency, age, gender, education, and Wealth Index); ^c^ Model adjusted for individual covariates and the social environment index; ^d^ Model adjusted for individual covariates, contextual covariate and all neighborhood scales. All models include city as fixed effect.

## Supplementary Materials

The following are available online at https://www.mdpi.com/1660-4601/17/23/8956/s1: Table S1: Number of items, Cronbach’s alpha, psychometric and ecometric properties of neighborhood scales, CAF Survey, 2016–2017, and Table S2: Odds ratios of self-rated health associated with the area level characteristics before and after adjustment for covariates, without imputation, CAF Survey, 2016–2017.
