# Supplementary material for: Physical Disorders and Poor Self-Rated Health in Adults Living in Four Latin American Cities: A Multilevel Approach
Source: Int J Environ Res Public Health. 2020 Dec 2;17(23):8956. doi: 10.3390/ijerph17238956 (PMC7730272; doi:10.3390/ijerph17238956)
Supplement: Supplementary file 1 [file ijerph-17-08956-s001.pdf]

International Journal of
Environmental Research and Public Health

Supplementary

Table S1. Number of items, Cronbach's alpha, psychometric and ecometric properties of neighborhood scales, CAF Survey, 2016-2017.

Scale	No. of Items in Scale	Cronbach's Alpha	ICC(%)	Reliability(%)
Physical Disorder	5	0.62	15.98	95.27
Social Disorder	5	0.76	18.74	96.44
Access to Services	6	0.65	30.08	97.52
Access to Leisure Spaces	3	0.58	19.44	96.34

*N*º = number; ICC = intra-neighborhood correlation coefficients.

Table S2. Odds ratios of self-rated health associated with the area level characteristics before and after adjustment for covariates, without imputation, CAF Survey, 2016-2017.

VARIABLES	Model 1[a]		Model 2[b]		Model 3[c]		Model 4[d]	
	OR (95%CI)	p-value	OR (95%CI)	p-value	OR (95%CI)	p-value	OR (95%CI)	p-value
**Physical Disorder**	**1.20 (1.08 - 1.32)**	**0.001**	**1.16 (1.04 - 1.29)**	**0.006**	**1.14 (1.02 - 1.27)**	**0.022**	**1.12 (0.99 - 1.28)**	0.075
**Social Disorder**	1.16 (1.04 - 1.29)	0.006	1.12 (1.01 - 1.25)	0.034	1.11 (0.99 - 1.23)	0.056	1.04 (0.92 - 1.18)	0.526
**Access to Services**	1.00 (0.90 - 1.11)	0.979	1.03 (0.93 - 1.15)	0.555	1.09 (0.97 - 1.22)	0.142	1.05 (0.91 - 1.20)	0.512
**Access to Leisure Spaces**	0.94 (0.84 - 1.05)	0.280	1.00 (0.89 - 1.12)	0.998	1.05 (0.93 - 1.18)	0.454	1.06 (0.92 - 1.22)	0.456

SD = standard deviation

N individual level = 3,362; N contextual level = 133

[a]Bivariate analyses between poor self-rated health and neighborhood scales.

[b]Model adjusted for individual covariates (length of residency, age, gender, education, and Wealth Index).

[c]Model adjusted for individual covariates and the social environment index.

[d]Model adjusted for individual covariates, contextual covariate and all neighborhood scales.
